# Protocol for the process evaluation of a cluster randomised controlled trial to determine the effectiveness and cost-effectiveness of independent pharmacist prescribing in care home: the CHIPPS study

**DOI:** 10.1186/s13063-020-04264-8

**Published:** 2020-05-29

**Authors:** Christine M. Bond, Richard Holland, David P. Alldred, Antony Arthur, Garry Barton, Linda Birt, Annie Blyth, James Desborough, Joanna Ford, Christine Handford, Helen Hill, Carmel M. Hughes, Vivienne Maskrey, Kate Massey, Phyo K. Myint, Nigel Norris, Fiona M. Poland, Lee Shepstone, Arnold Zermansky, David Wright, On behalf of the CHIPPS Team, Laura Watts, Joanna Williams, Bronwen Harry, Jeanette Blacklock, Caroline Hill, Frances Johnston, Jacqueline Inch, Frances Notman, Lindsay Dalgarno, Amrit Daffu O’Reilly, Mairead McGrattan

**Affiliations:** 1 Institute of Applied Health Sciences, School of Medicine, Medical Sciences & Nutrition, University of Aberdeen, Foresterhill, Aberdeen, Scotland, UK; 2 Leicester Medical School, University of Leicester, Leicester, UK; 3 School of Healthcare, Baines Wing, University of Leeds, Leeds, UK; 4 School of Health Sciences, Faculty of Medicine and Health Sciences, University of East Anglia, Norwich, UK; 5 Norwich Medical School, University of East Anglia, Norwich, UK; 6 School of Health Sciences, University of East Anglia, Norwich, UK; 7 School of Pharmacy, University of East Anglia, Norwich, UK; 8 Addenbrookes Hospital, Cambridge, UK; 9 Norfolk & Suffolk Primary and Community Care Research Office, hosted by South Norfolk Clinical Commissioning Group, Norwich, UK; 10 Athena Care Homes, Unit 2 Rima House, A13 Approach, Ripple Road, Barking, Essex, IG11 0RH UK; 11 School of Pharmacy, Queen’s University Belfast, Belfast, Northern Ireland, UK; 12 School of Education & Lifelong Learning University of East Anglia, Norwich, UK; 13 School of Healthcare, University of Leeds, Leeds, UK

**Keywords:** Older people, pharmacist prescribing, care homes, polypharmacy, randomised controlled trial

## Abstract

**Background:**

Prescribing, monitoring and administration of medicines in care homes could be improved. A cluster randomised controlled trial (RCT) is ongoing to evaluate the effectiveness of an independent prescribing pharmacist assuming responsibility for medicines management in care homes compared to usual care.

**Aims and Objectives:**

To conduct a mixed-methods process evaluation of the RCT, in line with Medical Research Council (MRC) process evaluation guidance, to inform interpretation of main trial findings and if the service is found to be effective and efficient, to inform subsequent implementation.

**Objectives:**

1. To describe the intervention as delivered in terms of quality, quantity, adaptations and variations across triads and time.
2. To explore the effects of individual intervention components on the primary outcomes.
3. To investigate the mechanisms of impact.
4. To describe the perceived effectiveness of relevant intervention components [including pharmacist independent prescriber (PIP) training and care home staff training] from participant [general practitioner (GP), care home, PIP and resident/relative] perspectives.
5. To describe the characteristics of GP, care home, PIP and resident participants to assess reach.
6. To estimate the extent to which intervention delivery is normalised among the intervention healthcare professionals and related practice staff.

**Methods:**

A mix of quantitative (surveys, record reviews) and qualitative (interviews) approaches will be used to collect data on the extent of the delivery of detailed tasks required to implement the new service, to collect data to confirm the mechanism of impact as hypothesised in the logic model, to collect explanatory process and final outcome data, and data on contextual factors which could have facilitated or hindered effective and efficient delivery of the service.

**Discussion:**

Recruitment is ongoing and the trial should complete in early 2020. The systematic and comprehensive approach that is being adopted will ensure data is captured on all aspects of the study, and allow a full understanding of the implementation of the service and the RCT findings. With so many interrelated factors involved it is important that a process evaluation is undertaken to enable us to identify which elements of the service were deemed to be effective, explain any differences seen, and identify enablers, barriers and future adaptions.

**Trial registration:**

ISRCTN17847169.

Date registered: 15 December 2017.

## Introduction

Medication management in care homes is suboptimal [1]. Despite various policy recommendations [2–7], the majority of care home residents continue to receive inappropriate medication, and a significant number of administration errors occur [8]. Many residents are on multiple medications, and there is overuse of psychotropic medicines, as well as more general concern such as lack of biochemical monitoring of high-risk drugs including methotrexate, azathioprine, amiodarone, warfarin etc. and lack of regular medication review [9]. Further, there is often poor communication between general practitioner (GPs), care home staff, community pharmacists, residents and their relatives, and a need for training of care home staff, many of whom may not have a theoretical understanding of the requirements for supply, storage, recording and administration of medicines. High staff turnover and the frailty of the resident population are further factors. It has been suggested that errors would be reduced if a single person took on overall responsibility for the medicines management in care homes. Our team proposed this should be a pharmacist independent prescriber (PIP) linked to the care home, and are undertaking a programme of work which has identified logistical and professional barriers to a PIP care home service, and devised solutions to address them [10], devised a training programme for the PIPs to ensure they have the requisite competencies to deliver the service, and identified provisional measures of outcome [11] in preparation for a definitive cluster randomised controlled trial (RCT) to compare the outcomes from the PIP service with usual care. A mixed-methods non-randomised feasibility study [12] confirmed the acceptability and feasibility of the processes for participant identification, recruitment and informed consent. The PIP service was found to be acceptable to all stakeholders and benefits were reported by GPs and care home staff. Appropriate outcome measures and tools were confirmed and minor refinements were made to the service specification and RCT protocol. The definitive cluster RCT is ongoing and will complete in May 2020. An internal pilot within the definitive trial has confirmed: (i) the feasibility of recruiting and randomising sufficient GP practices, PIPs, care homes and residents; (ii) the availability of data for primary outcome at 3 months; (iii) confirmation that there are no intervention-related safety concerns and (iv) researcher blinding and unblinding [12].

The definitive RCT was approved by ethics committees in England and Scotland, and registered with the ISRCTN registry (registration number ISRCTN 17847169). A protocol paper (protocol version 5 1.7.18) for the main trial, following SPIRIT guidance, has been submitted to BMC *Trials* [13]. This current article describes the protocol for the Process Evaluation Protocol that accompanies the definitive RCT; it follows the 2014 guidance on process evaluations [14, 15] and details are in Process Evaluation Protocol version 4 11.12.18 available from the authors on request.

## Causal assumptions

The definitive RCT is evaluating the efficacy and efficiency of implementing and delivering a PIP service and comparing outcomes for care home residents who receive the PIP service with outcomes for residents who continue to receive usual care [13]. In total 44 GP/PIP/care home groups (subsequently referred to as triads) and 880 patients will be included in four geographical areas of East Anglia, Leeds, North East Scotland and Northern Ireland. As noted above, the problem is that medicines management in care homes is suboptimal and complex, because the frailty and comorbidities of residents means many are on multiple medications. A logic model was developed (Additional file 1: Appendix 1) to demonstrate how the proposed PIP service could address the various issues. The PIP intervention is described below.

### The intervention

At intervention care home(s), PIPs working in collaboration with the relevant GP(s), will assume responsibility for the medicines management of a mean of 20 care home residents living in one or more care homes associated with the GP practice. To ensure competency in professional role and study procedures, the recruited PIPs are qualified independent prescribers, and have attended a 2-day face-to-face training programme, which included an overview of the trial design, project delivery, and preparation for the role; the service specification and completion of the Pharmaceutical Care Plans (PCPs). This was followed by time to develop relationships with medical practices (for those unfamiliar with the practice), care homes and community pharmacists, including performing medication reviews with GPs and observing medication administration by care staff, before final sign-off by clinically qualified professionals independent of the research team.

A service specification for the PIPs has been developed iteratively in the earlier stages of the project, and is attached (Additional file 1: Appendix 2). In summary, it includes:

- reviewing a participating resident’s medication and developing and implementing a PCP
- assuming prescribing responsibilities
- supporting systematic ordering, prescribing and administration processes within each care home, GP practice and supplying pharmacy where needed
- providing training to staff in care home and GP practice
- communicating with GP practice, care home, supplying community pharmacy and study team

### The control arm

At each control care home, medicine management is according to usual practice, in which the GP(s) has responsibility for the medicines management of care home residents living in one or more care homes associated with the GP practice. Pharmacy provision is also according to usual practice in that area.

### The CHIPPS RCT process evaluation

The aims of the process evaluation, described in this article are informed by the logic model and the stages of the Medical Research Council (MRC) process evaluation framework [14, 15]. These are listed below.

1. To describe the intervention as delivered in terms of quality, quantity, adaptations and variations across triads and time (Table 1).
2. To explore the effects of individual intervention components on the primary outcomes (Tables 2 and 3).
3. To investigate the mechanisms of impact (Table 2).
4. To describe the perceived effectiveness of relevant intervention components (including PIP training and care home staff training) from participant (GP, care home, PIP and resident/relative) perspectives (Tables 2, 3 and 4).
5. To describe the characteristics of GP, care home, PIP and resident participants to assess reach (Table 4).
6. To estimate the extent to which intervention delivery is normalised among the intervention healthcare professionals and related practice staff (Table 4).
7. If the service is found to be effective and efficient, to inform subsequent implementation.

**Table 1:** Implementation tasks and data collected as part of process evaluation

Task	Aim (what is being assessed)	Data collected	Data source
Provide training for PIPs	Effectiveness of training	PIP views on training	Post- training feedback forms (at end of 2-day training session)
			PIP interview PIP questionnaire
		Competency	Competency assessments (feedback from independent assessors)
			Appropriateness of PCPs (20% sample; Additional file 1: Appendices 3,4)
			Views of stakeholders (interviews)
PIP delivery of the intervention	Fidelity to intervention	Services provided and frequency with which provided	PIP activity logs
			Number of pharmaceutical care plans
			PIP questionnaire
		Quality of medication review	Review of 20% of pharmaceutical care plans

*PCP* Pharmaceutical Care Plan; *PIP* pharmacist independent prescriber

**Table 2:** Mechanism of impact and data collected as part of process evaluation

Impact	Mechanism of impact	Data collected	Data source
Medication changes identified	PIP medication review	Recommendations for change and rationale	Pharmaceutical care plans
			PIP interview PIP questionnaire
Medication changes made	PIP prescribing	Total no. medications per patient at baseline and 6 months	Pharmaceutical care plans
			GP records
		No. medications stopped per patient at 6 months	Pharmaceutical care plans
			GP records
		No. medications started per patient at 6 months	Pharmaceutical care plans
			GP records
		No. medications amended, e.g. dose change, formulation change	Pharmaceutical care plans
			GP records
		No. antipsychotics/psychotropics prescribed at baseline and 6 months	Pharmaceutical care plans
			GP records
		Categorised description of drugs changed, stopped, started	Resident medical records
Biochemical monitoring	PIP medication review	Recommendations made for biochemical monitoring	Pharmaceutical care plans
Medication errors	PIP medication review	Number of prescribing, dispensing and administration errors	Pharmaceutical care plans
			GP records
Non-patient-facing activities improved, e.g. medication storage advice	PIP support for care home	Services provided and frequency	PIP activity log
		Views on usefulness of services	Care home staff interviews
			PIP interview PIP questionnaire
Better/tailored training for staff	PIP training for care home staff	Training provided and frequency	PIP activity log
		Views on usefulness of training	Care home staff interviews
			PIP interview PIP questionnaire
Quality of communication between care home, GP and community pharmacy improved	PIP input into improved communication	Views of care home staff	Care home staff interviews
		Views of GPs	GP interview
		Views of PIPs	PIP interview PIP questionnaire

*GP* general practitioner; *PIP* pharmacist independent prescriber

**Table 3:** Outcomes and data collected as part of process evaluation

Aim	Outcome	Data collected	Data source
To improve quality of care for those over 65 years old resident in care homes	Falls	Fall rate per person at 3 months	Care home falls record
		Fall rate per person at 6 months	Care home falls record
	Quality of life	Self-reported quality of life	Face-to-face self-reported EQ-5D-5 L (only applicable for participants with capacity) at baseline, 3 months and 6 months
		Carer-assessed quality of life	Proxy EQ-5D-5 L (quality of life) at baseline, 3 months and 6 months
	Physical functioning	Carer-assessed physical functioning	Proxy Barthel Index (physical functioning) at baseline and 6 months
	Health service utilisation and associated costs	Costs of care (medication, healthcare team contacts, monitoring and tests)	GP records at baseline and 6 months
	DBI	Calculate DBI based on medications	GP records at baseline and 6 months
To assess intervention safety	Mortality	Information on numbers dying and time to death.	Monthly call to care homes
	Hospitalisations (Note: not always a negative marker of safety)	Information on numbers hospitalised	Monthly call to care homes
	Global view^a^	Perceptions of GPs	GP interview
		Perceptions of care home staff	Care home staff interviews
		Perception of residents/consultee/WPOA	Resident/consultee/WPOA interviews
		Perceptions of PIPs	PIP interview
	Adverse events^a^	New drug related symptoms	Stakeholder feedback using standard template
	Serious adverse events^a^	See hospitalisations/deaths	Monthly call to care homes
	Sudden unexpected serious adverse events^a^	See hospitalisations/deaths	Feedback from GPs/independent medical assessor on causal link with PIP intervention

*DBI* Drug Burden Index; *EQ-5D-5 L* EuroQoL five-dimension, five-level questionnaire; *GP* general practitioner; *PIP* pharmacist independent prescriber; *WPOA* Welfare Power of Attorney

^a^ Other than those noted, these are also primary and secondary outcomes for main trial and will be compared across groups

**Table 4:** Contextual factors collected as part of process evaluation

Contextual factor		Data collected	Data source
Barriers to delivering the intervention		Feedback from stakeholders	Care home staff interview
			GP interview
			PIP interview
			NoMAD [16] survey to GPs/PIPs and care home staff
			Other anecdotal feedback
Facilitators to delivering the intervention		Feedback from stakeholders	Care home staff interviews
			GP interview
			PIP interview
			NoMAD [16] survey to GPs/PIPs and care home staff
			Other anecdotal feedback
Site and participant factors	Inter PIP variation	Competency	Variation in outcomes
			Review of PCPs for both safety and missed opportunity
			GP interview
			Care home interviews
		Employment status	Baseline PIP questionnaire
		Qualifications	Baseline PIP questionnaire
	Inter-site variation	Care home factors	Baseline care home survey
		Resident factors	Baseline resident data
	Inter-location variation	Views of researchers	Meeting minutes
Normalisation of intervention into routine practice	Actions taken by participants to ensure the intervention works	Coherence (Making sense of the service)	NoMAD survey [16] to PIPs, care home staff, GPs
			GP interview Care home staff interviews
			PIP interview
		Cognitive participation (Engaging with the service)	NoMAD [16] survey to PIP, care home staff, GPs
			Interviews (GP and care home staff)
			PIP interview
		Collective action (delivering the service/responding to the service)	NoMAD [16] survey to PIP, care home staff, GPs
			GP interview Care home staff interviews
			PIP interview
		Reflexive monitoring (appraising and reviewing the service)	NoMAD [16] survey to PIP, care home staff, GPs
			GP interview Care home staff interviews
			PIP interview

*GP* general practitioner; *PIP* pharmacist independent prescriber

## Methods

### Design

In line with the MRC guidance on process evaluation [14, 15] this mixed-methods process evaluation using qualitative and quantitative approaches will collect data on implementation of the intervention, mechanisms of impact, outcomes and contextual factors. The tasks, aims, data and data source for each of these are summarised in Tables 1, 2, 3, 4. All data is collected, from intervention arm participants only, after the 6-month study period is completed for an individual participant.

### Implementation

Data will be collected on the effectiveness of the training, and the services delivered by the PIPs (see Table 1 below) to provide an understanding of whether the PIPS were adequately prepared for the role and the fidelity with which it was delivered.

### Mechanism of impact

Data will be collected to confirm the mechanism of impact of the intervention in achieving the desired aim of improved patient quality of care (Table 2). This section draws particularly on the logic model and hypotheses for addressing the highlighted issues. Data will only be collected from the intervention group to see if any observed differences in outcomes between the groups can be explained by different components of the PIP service. None of these happen in the control (usual care) group.

### Outcomes

The outcomes that are collected and which will be used in the process evaluation are described in Table 3. The selected outcomes are those where there is a clear link to the intervention proposed and where they inform the process.

### Contextual factors

Any contextual factors identified which might have affected the delivery and impact of the intervention are described in Table 4. This information may include factors related to individual personnel and organisations as well as macro-level issues such as Care Quality Commission requirements or head office requirements.

### Data collection methods

The following text refers to the data sources identified in Tables 1–4 .

### Quantitative

Data sources related to training and pharmacist competency

- *Training feedback*: At each PIP training event, PIPs are asked to complete a feedback form at the end of the 2-day face-to-face session
- *Pre-intervention competency*: Following the training, PIPs submit their competency framework to one of the study competency assessors who discuss these with the PIP and signs them off as ‘fit to practise’ as a CHIPPS PIP, prescribes further training or that they are not competent to deliver the study
- *Review of PCPs*: Following an agreed process (Additional file 1: Appendices 3 and 4) a random 20% of PCPs are reviewed for appropriateness by study team members who are specialists in care of the elderly. Whilst this process is primarily about safety, the assessment templates also capture data on missed opportunities.

Data sources related to activity

- *PIP activity log:* Intervention PIPs are asked to keep an activity log of their daily activity detailing the time spent on tasks as listed in the service specification (Additional file 1: Appendix 2)
- *PIP survey*: Following each phase, intervention PIPs will be asked to complete a short questionnaire asking about their experiences and the extent to which they delivered aspects of the intervention focusing especially on non-medication review aspects of the service specification (the NoMAD survey [16]).

Data sources related to prescribing

Most of the prescribing-associated data is collected as part of the main trial processes to assess effectiveness and efficiency of the intervention (GP records, healthcare utilisation, falls records, hospitalisations and deaths) and processes are detailed in the main trial protocol (version 5 1.7.18). The following lists additional data collected as part of the process evaluation

- *Adverse events*: Adverse events which are not deemed serious are reported using a standard template emailed to the Clinical Trials Unit. All study participants with a professional role (PIP GP, GP staff and care home staff) are made aware of this template and are asked to use this facility if they suspect any adverse event, whether or not there is a perceived causal relationship with the intervention
- *PCPs*: these are completed by the PIP as a clinical record of their actions including the rationale for these. Data extraction from these will inform the details of medication changes that underpin the global measures such as total number of medicines, British National Formulary categories most involved in changes, and overall Drug Burden Index (DBI). They will also include information on homely remedies and medications available from pharmacies (P medicines) and other retail outlets (general sale list medicines) which could result in therapeutic duplication

Data sources related to variability

Variability may be due to inherent non-modifiable differences across participating organisations, sites and individuals, or to the way the CHIPPS service has been delivered or normalised. The former will be explored using subgroup analyses and the latter by general estimating equations (GEEs) and applying normalisation process theory (NPT) via a NoMAD [16] survey to all participating GPs, PIPS and care home staff at the end of each phase. These are described below.

- *Subgroup analyses:* The following subgroup analyses will be conducted.
  i. Comparison of intervention effect by care home types, i.e. nursing versus residential.
  ii. Comparison of intervention effect by the employment status of the PIPs, i.e. those PIPs who were previously employed, and therefore had an established working relationship, with the study GP practice, and those who were not).
  For both of the above an interaction term (between treatment and subgrouping factor) will be added to the primary model and formally tested for a non-zero value.
- *GEEs:* Any effect of the PIP intervention is likely to be mediated through a decrease in the DBI. This will be tested using a GEE, adjusting for group membership (this is in order to remove any effect of the PIP intervention on falls mediated via a different causal route).
- *NoMAD survey* [16]: The NoMAD survey is an implementation measure based on the Normalisation Process Theory (NPT) [17, 18]. The survey form includes preliminary demography and general questions about experiences and satisfaction followed by four sections each relating to one of the NPT domains of coherence, cognitive participation, collective action and reflexive monitoring.

### Qualitative

Feedback from all stakeholders on their experiences and views of the intervention is a core part of this process evaluation. This will help contextualise the intervention and increase understanding of the process of implementation and any variation between sites and stakeholders.

#### Interviews

At the end of each phase of the intervention a purposive sample of up to three of each of GPs, care home managers, staff, residents and relatives (if available) in each of the four geographical areas will be invited to take part in a semi-structured interview. Sampling will be based on a maximum variation sample to reflect differences in site and PIP characteristics, e.g. PIP employment status, previous PIP experience, demographic profile of care home residents, rural or urban location. All PIPS will be invited to take part in an interview.

Interviews will be guided by a topic guide (Additional file 1: Appendix 5), developed from the qualitative outputs from the earlier non-randomised feasibility study [12]. Topics will include participants’ views of the PIP service implementation and delivery, communication between staff, perceived effectiveness of the intervention and the identification of any unintentional consequences. All aspects of the service will be probed and there will be specific probes for unforeseen effects to understand whether anything else about the service impacted positively or negatively on patient care or cost-effectiveness. In addition to the above topics, the PIP interview will explore their perception of the training programme and its utility.

#### Conduct and analysis

All participants invited to interview will be given an information sheet and consent form prior to participation. Ideally, interviews will be held face to face at a location of the interviewee’s choice, but virtual modes will be considered for logistical reasons. All proceedings will be audio recorded and transcribed verbatim. Thematic analysis will draw on the NPT framework, but an inductive approach will enable recognition of unexpected emergent themes. Data will be managed in NVIVO.

#### Documentary evidence

Minutes of meetings will provide researcher-reported information on barriers, facilitators and other confounding factors that may have affected delivery of the trial, e.g. (recruitment challenges, reach).

### Data integration/synthesis

Once all process and main trial outcomes are reported, all the data sets (qualitative and quantitative) will be integrated [19] using a triangulation approach to consider agreement, partial agreement, silence and dissonance across the findings. This will identify relevant actions, and clarify and relate causal pathways to experiences, providing an enriched means to explain unexpected outcomes, and identify optimal intervention contexts. Should the main RCT findings suggest the CHIPPS service is effective and efficient, the process evaluation will inform recommendations for implementation into routine services. The process evaluation will also be interrogated to understand reasons why the intervention has not been successful, including variable success rates in different sites.

## Discussion

This detailed mixed-method process evaluation will provide an in-depth understanding of the interactions, barriers and facilitators which underpin the main study quantitative findings. For example, it will provide evidence of the effectiveness of the training in preparing the PIPs to deliver their role, show to what extent the mechanism of impact – improved medication management – has been implemented and the barriers and facilitators that have been encountered. Learning from these will enable decisions to be made about future roll-out of the PIP service. Further, if the main trial does not demonstrate that there has been an improvement in patient outcomes, it will allow an informed judgement to be made as to whether the service is wrong in principle or whether its implementation has been suboptimal. Our findings will be especially pertinent and timely as PIPs are already being introduced into care homes for roles such as we are evaluating. The systematic and comprehensive approach that is being adopted is in line with the MRC guidance on process evaluations [14, 15]. It will ensure data is captured on all aspects of the study, and allow an understanding of the implementation of the service, confirm its mechanism of impact, explore secondary, possibly explanatory, outcomes and any contextual factors. In summary, with so many interrelated factors involved it is important that a process evaluation is undertaken to enable identifyimg which elements of the service were deemed to be effective and explain any differences seen whilst also identifying adaptions, enablers and barriers.

### Trial status

Resident recruitment for the RCT began in February 2018 and will continue until October 2019. Recruitment for the process evaluation started spring 2019 and will continue until June 2020.

## Supplementary information

**Additional file 1: Appendix 1.** CHIPPS Logic Model v8, 31/10/2018 CHIPPS Logic Model v8, 31/10/2018. **Appendix 2.** Care Homes Independent Pharmacist Prescribing Study (CHIPPS) Service Specification. **Appendix 3.** Review of Pharmaceutical Care Plans. **Appendix 4.** CHIPPS PCP reviews – reporting template. **Appendix 5.** Topic guides for interviews and focus groups.

## Data Availability

Requests for access to the data will be considered, and approved in writing where appropriate, after formal application to the Trial Management Committee/Programme Steering Committee [13]. Considerations for approving access are documented in the Project Management Group/Programme Steering Committee Terms of Reference [13].
