# Supplementary material for: Protocol for the process evaluation of a cluster randomised controlled trial to determine the effectiveness and cost-effectiveness of independent pharmacist prescribing in care home: the CHIPPS study
Source: Trials. 2020 May 29;21:439. doi: 10.1186/s13063-020-04264-8 (PMC7257128; doi:10.1186/s13063-020-04264-8)
Supplement: Supplementary file 1 — Additional file 1: Appendix 1. CHIPPS Logic Model v8, 31/10/2018 CHIPPS Logic Model v8, 31/10/2018. Appendix 2. Care Homes Independent Pharmacist Prescribing Study (CHIPPS) Service Specification. Appendix 3. Review of Pharmaceutical Care Plans. Appendix 4. CHIPPS PCP reviews – reporting template. Appendix 5. Topic guides for interviews and focus groups. [file 13063_2020_4264_MOESM1_ESM.docx]

**Appendix 1 CHIPPS Logic Model v8, 31/10/2018 CHIPPS Logic Model v8, 31/10/2018**

**The problem Context Inputs Outputs Process outcomes Clinical outcomes Outputs**

Complexity of patients - frailty & co morbidities management

Regular review of systems & procedures; protocols, optimising & monitoring

Medicines management in Care Homes (CHs) suboptimal

No single person responsible

No regular individual patient medication review

Communication gaps between

GPs, CHs, CPs, Residents, Relatives

CH staff workload and experiences einces

Staff satisfaction

PIP responds to medicines information queries

PIP gives information to residents **&** relatives

PIP training

& accreditation

Medicines reconciliation

Training CH staff on medicines’ administration and use

PIP Develop & implement pharmaceutical care plan

Pharmacist PIP roles increasing and supported by policy makers

CH staff, GPs, resid’s/relatives want medicines support cf turf wars

PIP satisfaction

GP satisfaction

GP workload and experiences

Interventions and rationale for them

Appropriateness of medicines

Reduced number of medicines/DBI

Reduction in errors

PIP workload and experiences experiences

Number of medicine requests

Knowledge gaps:

CPs: Of older people’s medicine.

CH staff:

i. Of medicines

ii. Of range of PIP skill and knowledge

Abbreviations:

ADE Adverse Drug Event

CCG Clinical Commissioning Groups

CH Care Home,

CP Community Pharmacist,

ETCs Excess Treatment Costs

GP General Practitioner,

PCT Primary care Team

PIP Pharmacist Independent Prescriber

QoL Quality of Life

Competency assured PIP

Clinical control e.g. BP, Cognitive ability, HBA1C

Prescribing optimisation

Enhanced communication

Stock holding optimisation

Administration optimisation

Resident and carer satisfaction

Mortality

Resident quality of life

Health & social care utilisation and costs

Adverse events e.g. falls, hospitalisation, mortality

**External (confounding) factors:**

- Leeds ETCs

- Other pharmacist schemes

- Recruiting people without capacity

**Assumptions:** Prescribing pharmacist can improve prescribing appropriateness and so can lower risk of ADEs and hospitalisations with:

- Expertise in medicines management

- Specialised pharmaceutical knowledge

Colour Key: System Pharmacist (PIP) Training Outcomes

PIP monthly authorisation repeat prescriptions

CH staff lack medicines management skills

## Appendix 2.

**Care Homes Independent Pharmacist Prescribing Study (CHIPPS) Service Specification**

1. **Service outline**

CHIPPS is a National Institute for Health Research (NIHR) programme grant to develop and deliver a cluster randomised controlled trial to determine the effectiveness and cost-effectiveness of making pharmacist prescribers part of a team working alongside care home staff and General Practitioners (GPs) in care homes for older people. CHIPPS will provide a Pharmacist Independent Prescriber (PIP) to review and optimise prescribing in recruited residents and facilitate and support cost effective evidence based prescribing and medicines management in care homes for older people.

1. **Aims and objectives**

The aim of the service is to improve health outcomes and wellbeing of care home residents and ensure medicines are prescribed and managed in a safe, effective and cost effective way.

In order to meet the stated aims recruited GP practices and care homes will work with a PIP who has demonstrated competency in care home medicines management and prescribing in older people. The PIP will be based at the GP practice, for the duration of the study, and will have developed an excellent working relationship with the GP practice and care home prior to commencing the service delivery. The service will run for a period of 6 months.

**3. Inclusion/exclusion criteria for service**

**3.1 Pharmacist Independent Prescriber (PIP)**

Inclusion criteria

- Registered as a pharmacist independent prescriber
- Following training can demonstrate competence to deliver service (See section 4)
- Ability to work flexibly and commit a minimum of 16 hours a month to deliver the service for three months

Exclusion criteria

- Substantive employment with the community pharmacy (branch/store) which supplies medicines to the care home with which the PIP would work

**3.2 Care home**

Inclusion criteria

- Care Quality Commission (CQC) registered specialism as caring for adults over 65
- Primarily caring for residents over 65 years

Exclusion criteria

- Care homes who receive additional medication focussed services with a visit frequency ≥ monthly
- Care homes which only provide carer or support remotely (They do not have carers on site 24 hours a day)
- Care homes which are currently under formal investigation with the Care Quality Commission (CQC) or equivalent body

**3.3 Residents**

Inclusion criteria

- Resident under the care of the participating GP practice
- Residents currently prescribed at least 1 medicine
- Residents or their appropriate representative who are/is able to provide informed consent/assent
- Permanent resident in care home (not registered for respite care/temporary resident)
- Residents must be 65 years or over

Exclusion criteria

- Residents who are currently receiving end of life care (equivalent to yellow (stage C) of the Gold Standards Framework prognostic indicator)
- Resident with additional limitations on their residence (e.g. held securely)
- Participating in another research study

**4. Service requirements**

**4.1. Recruitment and employment of the Pharmacist Independent Prescriber (PIP)**

- Initial identification and recruitment of the PIP will be conducted by the CHIPPS management committee
  - The PIP will require:
    - Excellent interpersonal, communication and IT skills
    - Familiarity with relevant GP software systems
    - Experience of providing prescribing and medicines management advice and support
    - Previous experience of working in GP practice environment
    - Be able to travel to site locations
    - A mobile phone to be contactable for the purposes of delivering this service
    - Appropriate indemnity insurance for prescribing
- PIP will be employed according to local arrangements and seconded to the relevant GP practice for the duration of the study and during training and competency assessment (see section 4.2)

**4.2 Training and competency assessment of PIP**

See Appendix 1

**4.3 PIP roles and responsibilities [NB: categorised as essential or not]**

The PIP will, where appropriate:

4.3.1 Review each resident’s medication and develop and implement a pharmaceutical care plan^^[[1]](#footnote-1)^^ (essential)

- Optimise prescribing ensuring clear indication and evidence base for each medication (taking into consideration national and local pathways, guidelines and formularies), informed by tools such as STOPP/START
- Minimise the potential for adverse effects
- Optimise the dose of all medication
- Co-ordinate appropriate monitoring and associated tests for all medicines and conditions
- Agree initial care plan with GP, care staff and resident (where appropriate)
- Document and maintain records relating to review and care plan in GP and care home records as appropriate

4.3.2 Prescribing (essential)

- Authorise repeat prescriptions
- Co-ordinate appropriate monitoring and associated tests for all medicines and conditions
- Deprescribe medicines according to agreed pharmaceutical care plan
- Document medication changes in GP and care home records and notify supplying pharmacy of all changes to medication within 24 hours
- Only initiate new medicines for existing diagnoses or for common ailments which can be managed with medicines classified by the Medicines and Healthcare products Regulatory Agency (MHRA) as Pharmacy (P) or General Sales List (GSL)
- Any additional areas of prescribing must be agreed and documented with the GP practice prior to prescribing (e.g. antibiotics for simple UTIs)

4.3.3 Communication (essential)

- Agree local protocols for communication with GP practice and care home prior to commencing service. This should include:
  - Process of communication and messaging
  - The location and expected level of detail of all PIP interventions in the medical records Process and communication of referrals for activities outside the competence of the PIP
- Inform supplying community pharmacy about service and role (prior start of service)
  - Communicate all changes in medication to supplying pharmacy
- Complete all documentation and recording of activities as required by the study team

4.3.4 Support systematic ordering, prescribing, and administration processes with each care home, GP practice and supplying pharmacy where needed: (undertaken at PIP’s discretion)

- Provide instructions on how to administer each drug
- Synchronise residents prescription quantities for monthly cycles
- Add or clarify directions for all medication where it is currently not clear
- Provide advice on repeat prescription ordering processes to:
  - Minimising missed items
  - Optimising quantities
- Optimise the use of homely remedies within the care home
- Reconcile resident medication following a transfer of care

4.3.5 Training provision (undertaken at PIP’s discretion)

- Review training needs of care home and GP practice and draft proposed training plan
- Provide training to care home staff on training needs basis from agreed list of potential topics/areas
- Provide guidance to relevant GP practice on training needs basis from agreed list of potential topics/areas

**4.4 Safe and effective service provision**

- PIP will be contactable and respond to messages within 24 hours (Monday - Friday)
- The PIP will establish a locally agreed protocol with the GP practice for referral/notification of all medicine related queries from CHIPPS participants to the PIP as appropriate (see 4.3.5)
- PIP will have full (read/write) access to GP record system to issues prescriptions and update records
- Where possible PIP will use remote access to update records when changes are made to GP held record
  - Where remote access is not feasible the PIP must update records within 24 hours of making a change
- PIP will have full (read/write) access to care home records to update records during all visits using appropriate local reporting systems
- The PIP will visit/contact the care home at least once a week
- The PIP will visit/contact the GP practice at least once a week
- Wherever possible, all annual leave should be agreed before the beginning of the study. A clear system for transfer of responsibility communicated to GP, care home and supplying pharmacy
- The PIP will work within the local prescribing formularies of GP practice and primary care organisation.
- The PIP will report and document all significant clinical events or near misses using local reporting procedures and study documentation.
- Ensure all records are aligned

Appendix 3 Review of Pharmaceutical Care Plans

## As part of the study procedures a random sample of PCPs are selected for review following the process defined in the WP6 protocol (version 5 dated 01.07.18 page 38) below (Box 1).

## The rationale is that it is important to check a PIP’s performance early on in the intervention in order to identify if any of the recruited PIPs, despite having satisfied the assessors of their competence, were not in fact prescribing appropriately. Therefore the sampling is done iteratively as blocks of 5 PCPs were completed, with a larger proportion of the first set of plans sampled, and an agreed procedure to follow for increased sampling should concerns be identified.

**Box:** **Guidance in study protocol for selecting the PCPs for review.**

Quality reviews of 20% randomly selected pharmaceutical care plans (PCP) will be undertaken for each PIP by a qualified medical practitioner.

- ROUND 1: review random 2 out of first 5 PCPs (as they are highest risk, essentially)
- ROUND 2: review random 1 out of next 5 PCPs (unless low level concerns, in which case 2 out of 5, moderate level concerns 3 out of 5, significant concerns after first five = Stop)
- ROUND 3: review random 1 out of next 5 PCPs (unless low level concerns remain – in which case 2 out of 5, if moderate level concerns remain consider Stopping at half-way point)
- ROUND 4: review 0 out of last 5 PCPs (assuming no concerns in previous, unless low level concerns remain in which case one should be sampled)

The exact details of the process are as follows.

- All PCPs ae identified by a unique numerical id.
- The appropriate number of random numbers is generated by the research team, using <https://www.random.org/integers/>.
- PIPs are asked to submit to the research team the anonymised PCPs corresponding to the random numbers generated together with any relevant documents needed to allow assessment of their decision.
- Grant holders who are specialists in care of the elderly medicine are assigned as reviewers.
- On receipt of the PCPs from PIPs, the research team emails them to either reviewer A or B.
- Using professional judgement the reviewers score the PCPs according to the following using a template (see Figure 1) with indicative descriptors illustrating level of harm, based on the National Coordinating Council for Medication Error Reporting and Prevention **(**NCC MERP) Taxonomy of Medical Errors^[[2]](#footnote-2)^ (which includes a severity rating/patient outcome measure).:
  - No concern
  - Low concern
  - Moderate concern
  - High concern
- In addition, two PCPs are independently reviewed by both reviewers A and B in each phase to provide an indication of inter-rater reliability. The reviewers are unaware which of the reviews they undertook, are being used for this check.
- Administrative feedback e.g. missing dates is provided to the PIPs for immediate correction.
- Clinical comments about the PCPs are provided at end of the intervention period unless serious concerns are identified (see Box).

**Appendix 4: CHIPPS PCP reviews – reporting template**

**CHIPPS PCP reviews – reporting template**

| **Date** |  | **Reviewer** |  |
| --- | --- | --- | --- |
| **PIP Number** |  | **PCP (Participant) Number** |  |

**Section 1**

| Do you have any general feedback on this PCP for the CHIPPS team? | Yes [ ] No [ ] |
| --- | --- |
| If Yes please expand  (*contributes to final CHIPPS process evaluation*) |  |
| Does this PCP suggest the intervention is being delivered to a level expected of a trained PIP? | Yes [ ] No [ ] Unsure [ ]  If **No** or **Unsure** please complete **Section 2 & Section 3** |

**Section 2 – type of concern**

**If NO or UNSURE, are your concerns**

| a. Because of a missed intervention (i.e. failure to amend an existing regime?) | Yes [ ] No [ ] |
| --- | --- |
| b. Because of an incorrectly made new prescribing/deprescribing decision | Yes [ ] No [ ] |

**Section 3 – Safety Assessment**

| **Are your concerns?** |  |  |
| --- | --- | --- |
| **A.**  **Low Level** | - PCP of a quality somewhat below the level expected of a trained PIP - no significant safety concern, no potential to cause harm to patient   Example: Missing patient details, incorrect terminology, missing fields | Yes [ ] |
| **B.**  **Moderate Level*** | - PCP of a quality well below the level expected of a trained PIP - some significant safety concerns that could cause temporary harm   Example: Omission of a drug after hospital discharge eg frusemide in a patient with heart failure | Yes [ ] |
| **C.**  **High Level*** | - PCP of a quality significantly below the level expected of a trained PIP - significant safety concerns with a potential to harm patient/need hospitalisation   Example: Use of NSAIDs without gastroprotection | Yes [ ] |
| **D.**  **Very High Level*** | - PCP of a quality significantly below the level expected of a trained PIP - significant safety concerns with a potential to seriously harm patient/result in death   Example: prescription error with insulin | Yes [ ] |

*** NB** PIPS will receive feedback if the review reveals concern levels **B, C and D**. Please complete more detailed feedback for these levels overleaf.

**Detailed feedback**

|  |
| --- |

**Appendix 5 Topic guides for interviews and focus groups**

# **CHIPPS WP6 QUAL Evaluation: Topic Guide – PIP 6 month training follow up focus group**

As the purpose of the focus group is to encourage a group conversation in which participants can feel confident in expressing their own views, the following topic guide is indicative. This means that the researcher may adapt it to suit the conversation style and preferences of the participants. As this is a group discussion it will be best to limit formal information-giving or structured questions. As PIPs will have had written information about the research purposes and for informed consent, this just need to be checked and confirmed. A focus group is not an interview and its purpose is to identify topics or relevance to group members.

As participants come in, understanding and consent will be confirmed.

|  | Introduction.   - 1. **Introduce yourself**   2. **Explain the purpose of the research** | We have developed a new service in which a specially trained ‘pharmacist independent prescriber’ (PIP) becomes part of the care home team, working alongside general practitioners. The aim is to improve how resident’s medicines are managed in a safe, effective and cost effective way. We hope this may improve resident’s care, wellbeing and health outcomes. We are evaluating this new service in a large multi-centre randomised controlled trial in different places in the United Kingdom.  The study is run by the universities of East Anglia, Aberdeen, Leeds and Queen's Belfast. It is funded by the UK National Institute for Health Research.  *Since participating in the training for ‘pharmacist independent prescribers (PIPs) ’ you have set up and have delivered the Care Home Independent Pharmacists Prescriber service in the trial,*  *We would like you to consider your overall role as a PIP providing this service and your experience in delivering this. We would also like your views on the Service Specification and Pharmaceutical Care Plan, your thoughts about the mentoring you received and how useful the personal development plan and training was in preparing you for this role and how this has impacted on your ability to set up and deliver this service.* |
| --- | --- | --- |
|  | The digital recorder  1. **Stress confidentiality** 2. **Set ground rules** | I would like to highlight the confidentiality of everything you tell me, and specifically that:   - *the recording will be deleted after being transcribed* - *you won’t be identified individually in any report.* - *all information will be anonymised.* - *we will not tell anyone else including your employer organisation, what you tell us as an individual.* - *likewise no other individual e.g. GP, care home staff, resident will be identifiable in any report.*   *We do need to remind you, however, that* if you do disclose anything which might identify a risk to yourself or to others, or a personal or professional offence, this would be shared with the relevant responsible authority. However we would tell you if we thought this were the case.   - All of your views are of value to us. There are no right or wrong answers, it is OK to disagree. - Please respect the views of others and not to talk over each other i.e. let people finish what they are saying. - Please ask me to clarify if the question isn’t clear. - We remind you not to share any personal or patient identifying information during this group - Additionally please do not discuss the content of the session outside the group |
|  | Ask if there are any questions |  |
|  | **Confirm consent** |  |

|  | **Stem question** | **Probes / follow ups** |
| --- | --- | --- |
| 1. | What was your experience of delivering the service? | - Any particular issues - Anything particularly good   (Interaction with GP/ care home staff/patients/ relatives  access to records/ information/routines /types of interventions)   - Acceptability to patients without capacity |
| 2. | What are your views on the Service Specification? | - Clarity? - Level of detail? - Inclusion/exclusion criteria - Service requirements? - Outcomes - Transitional arrangements |
| 3. | What are your views on the study documentation including: Pharmaceutical Care Plan, PIP log and resource use data? | - Ease of use - Anything missing/ not needed - Fit for purpose? |
| 4. | What were your thoughts on the role of the mentor? | - Positive - Negative - Areas for improvements |
| 5. | What was your experience of developing your own personal development plan with your mentor? | - Went well/ not so well - Mentor support/ time scale for completion - Accessing training on the identified development areas - General acceptability |
| 6. | What do you think about the competency framework assessment? | - Comprehensive? - Easy/hard to demonstrate achievement? |
| 7. | What do you think about the assessor signing you off? | - Useful / difficult discussion? - Appropriate? - Alternative suggestions? - Process fit for purpose? |
|  | **Stem question** | **Probes / follow ups** |
| 8. | What are your thoughts on the impact of training you received? | Any gaps-   - *preparation for role,* - *underpinning knowledge,* - *competency framework,* - *relationship building* - *personal development skills* |
| 9. | Now that you have been delivering the service for 3 months which particular elements of the training do you think have been most useful? | - Why? - Relevance - Time spent on this - Delivery |
| 10. | Which particular elements of the training do you think have been least useful in your role, delivering the service? | - Should we continue to provide this element of the training? - How should this element of training be delivered? - What should we do to improve these elements of training? |
| 11. | Is there anything that should have been included in the training that wasn’t? | - How would you suggest that we train pharmacists in that? - Is there existing training available, that you are aware of, to meet this - Need? - When should this training be provided? |
| 12. | It is important to us that across the four areas, all PIPs provide a similar service. What would be the best way for this to be achieved? | - Training? - Checklist? |
| 13. | In what ways could you contribute to the training of the next cohort of Pharmacist Independent Prescribers? | - Mentor? - Helping to deliver educational package locally? |
| 14. | Any final comments? |  |
| 15. | Thank you for taking part in this focus group | |

# **CHIPPS WP6 QUAL Evaluation: Topic Guide for stakeholder (Care Home Manager) interview at 6 Months**

As the purpose of this interview is to encourage a conversation in which the participant can feel confident in expressing their own views, the following topic guide is indicative. This means that the interviewer may adapt it to suit the conversation style and preferences of the participant.

|  | Introduction.   1. **Introduce yourself** 2. **Explain the purpose of the research** | We have developed a new service in which a specially trained ‘pharmacist independent prescriber’ (PIP) becomes part of the care home team, working alongside general practitioners. The aim is to improve how resident’s medicines are managed in a safe, effective and cost effective way. We hope this may improve resident’s care, wellbeing and health outcomes. We are evaluating this new service in a large multi-centre randomised controlled trial in different places in the United Kingdom.  The study is run by the universities of East Anglia, Aberdeen, Leeds and Queen's Belfast. It is funded by the UK National Institute for Health Research.  You have had a PIP working with you and your team providing this service in partnership with the GP practice to your residents for the last 6 months. In this interview we would like your views on the service and in particular your thoughts on the implementation and acceptability of the service as well as your perceptions of your working relationship with the PIP. |
| --- | --- | --- |
|  | The digital recorder  1. **Stress confidentiality** 2. **Set ground rules** | Can I check that you read and understood the participant information for this call? I would like to highlight the confidentiality of everything you tell me, and specifically that:   - *the recording will be deleted after being transcribed* - *you won’t be identified individually in any report.* - *all information will be anonymised.* - *we will not tell anyone else including your employer organisation, what you tell us as an individual.* - *likewise no other individual e.g. GP, care home staff, resident will be identifiable in any report.*   *We do need to remind you, however, that* if you do disclose anything which might identify a risk to yourself or to others, or a personal or professional offence, this would be shared with the relevant responsible authority. However we would tell you if we thought this were the case. |
|  | Ask if there are any questions |  |
|  | **Confirm consent** |  |

|  | **Stem question** | | | **Probes / follow ups** |
| --- | --- | --- | --- | --- |
| Part 1 | Views of the intervention | | |  |
| 1. | Overall what are your views of the PIP service? | | | - New contributions - New problems |
|  |  | | Views on changing skill mix in the primary care team  Views on changing skills mix in the care home team | - Diversity in team(s) - Challenges - Issues in managing |
| 2. | How has the PIP service impacted on your work load? (positively/negatively) | | | - Time - Referrals - Tests - Medication changes - Medication reviews - Repeat prescriptions - Medication administration processes - Record keeping - Medication storage - Interactions with GPs - Interaction with community pharmacist - Number of pharmaceutical advisory visits (i.e. not including PIP) |
| 3. | In what ways has the PIP service affected patient care? positively/negatively) | | | - Examples - Acceptability for patients without capacity? |
| 4. | Have you or your staff received any additional training since the introduction of the PIP service? | | | - Impact? |
| Part 2 | Implementation | | |  |
| 5. | What barriers if any to implementing this service? | | |  |
|  |  | *How could we overcome any barriers?* | |  |
| 6. | What did you see as facilitating the service to be implemented? | | |  |
| Part 3 | Working relationship | | |  |
| 7. | What is your view of your working relationship with the PIP? | | | - Good relationship areas - Difficult relationship areas |
|  |  | *How can your working relationship with the PIP be improved?* | |  |
| 8. | What is your view of your staffs’ relationship with the PIP? | | | - Good relationship areas - Difficult relationship areas |
|  |  | *How can their working relationship with the PIP be improved?* | |  |
| 9. | How has your working relationship with the PIP affected your relationship with your patients? | | | - New contributions   New problems |
| 10. | Where did communication issues arise between you and the other team members involved in the PIP service, and what were they? | | | - PIP - Community Pharmacist - Primary care Pharmacist - District Nurse - GP Practice |
| 11. | How do you think communication issues may have affected patient care? | | | - Examples |
| Part 4 | Acceptability | | |  |
| 12. | What aspects of the PIP service went well from your perspective? | | | - Time commitment - Paper work - Issues raised/resolved |
| 13. | What aspects of the PIP service went less well from your perspective? | | | - Time commitment - Paper work - Issues raised/resolved |
|  |  | *How could we improve these?* | |  |
| 14. | What elements of the service did you like best? | | |  |
| 15. | Overall, how satisfied are you with the service provided by the PIP? | | |  |
| 16. | Would you like the service to continue? | | | - In its current from - In a revised form - Not at all |
| Part 5 | Study procedures | | |  |
| 17. | Recruitment and participation | | | - Acceptability for residents with/without capacity - Time commitment - Paper work |
| 18. | Any final comments | | |  |
| 19. | Thank you for taking part in this interview | | | |

# **CHIPPS WP6 QUAL Evaluation: Topic Guide for stakeholder (Care Home Staff) interview at 6 Months**

As the purpose of this interview is to encourage a conversation in which the participant can feel confident in expressing their own views, the following topic guide is indicative. This means that the interviewer may adapt it to suit the conversation style and preferences of the participant.

|  | Introduction.   1. **Introduce yourself** 2. **Explain the purpose of the research** | We have developed a new service in which a specially trained ‘pharmacist independent prescriber’ (PIP) becomes part of the care home team, working alongside general practitioners. The aim is to improve how resident’s medicines are managed in a safe, effective and cost effective way. We hope this may improve resident’s care, wellbeing and health outcomes. We are evaluating this new service in a large multi-centre randomised controlled trial in different places in the United Kingdom.  The study is run by the universities of East Anglia, Aberdeen, Leeds and Queen's Belfast. It is funded by the UK National Institute for Health Research.  You have had a PIP working with you and your team providing this service in partnership with the GP practice to your residents for the last 6 months. In this interview we would like your views on the service and in particular your thoughts on the implementation and acceptability of the service as well as your perceptions of your working relationship with the PIP. |
| --- | --- | --- |
|  | The digital recorder **3. Stress confidentiality**  **4. Set ground rules** | Can I check that you read and understood the participant information for this call? I would like to highlight the confidentiality of everything you tell me, and specifically that:   - *the recording will be deleted after being transcribed* - *you won’t be identified individually in any report.* - *all information will be anonymised.* - *we will not tell anyone else including your employer organisation, what you tell us as an individual.* - *likewise no other individual e.g. GP, care home staff, resident will be identifiable in any report.*   *We do need to remind you, however, that* if you do disclose anything which might identify a risk to yourself or to others, or a personal or professional offence, this would be shared with the relevant responsible authority. However we would tell you if we thought this were the case. |
|  | Ask if there are any questions |  |
|  | **Confirm consent** |  |

|  | **Stem question** | | **Probes / follow ups** |
| --- | --- | --- | --- |
| Part 1 | Views of the intervention | |  |
| 1. | What involvement have you had with the PIP service? | |  |
| 2. | Overall what are your views of the PIP service? | | - New contributions - New problems |
|  |  | Views on changing skill mix in the primary care team  Views on changing skills mix in the care home team | - Diversity in team(s) - Challenges - Issues in managing |
| 3. | How has the PIP service impacted on your work load? (positively/negatively) | | - Time - Referrals - Tests - Medication changes - Medication reviews - Repeat prescriptions - Medication administration processes - Record keeping - Medication storage - Interactions with GPs - Interaction with community pharmacist |
| 4. | In what ways has the PIP service affected patient care? positively/negatively) | | - Examples - Acceptability for patients without capacity? |
| 5. | Have you received any additional training since the introduction of the PIP service? | | - Impact? |
| Part 2 | Implementation | |  |
| 6. | What barriers if any to implementing this service? | |  |
|  |  | *How could we overcome any barriers?* |  |
| 7. | What did you see as facilitating the service to be implemented? | |  |
| Part 3 | Working relationship | |  |
| 8. | What is your view of your working relationship with the PIP? | | - Good relationship areas - Difficult relationship areas |
| 9. |  | *How can your working relationship with the PIP be improved?* |  |
| 10. | How has your working relationship with the PIP affected your relationship with your patients? | | - New contributions   New problems |
| 11. | Where did communication issues arise between you and the other team members involved in the PIP service, and what were they? | | - PIP - Community Pharmacist - Primary care Pharmacist - District Nurse - GP Practice |
| 12. | How do you think communication may have affected patient care?  (positively/ negatively) | | - Examples |
| Part 4. | Acceptability | |  |
| 13. | What aspects of the PIP service went well from your perspective? | | - Time commitment - Paper work - Issues raised/resolved |
| 14. | What aspects of the PIP service went less well from your perspective? | | - Time commitment - Paper work - Issues raised/resolved |
|  |  | *How could we improve these?* |  |
| 15. | What elements of the service did you like best? | | - Patient care |
| 16. | Overall, how satisfied are you with the service provided by the PIP? | |  |
| 17. | Would you like the service to continue? | | - In its current from - In a revised form - Not at all |
| 18. | Any final comments? | |  |
| 19. | Thank you for taking part in this interview | | |

# **CHIPPS WP6 QUAL Evaluation: Topic Guide for stakeholder (Generic -other stakeholders) interview at 6 Months**

As the purpose of this interview is to encourage a conversation in which the participant can feel confident in expressing their own views, the following topic guide is indicative. This means that the interviewer may adapt it to suit the conversation style and preferences of the participant.

|  | Introduction.   - 1. **Introduce yourself**   2. **Explain the purpose of the research** | We have developed a new service in which a specially trained ‘pharmacist independent prescriber’ (PIP) becomes part of the care home team, working alongside general practitioners. The aim is to improve how resident’s medicines are managed in a safe, effective and cost effective way. We hope this may improve resident’s care, wellbeing and health outcomes. We are evaluating this new service in a large multi-centre randomised controlled trial in different places in the United Kingdom.  The study is run by the universities of East Anglia, Aberdeen, Leeds and Queen's Belfast. It is funded by the UK National Institute for Health Research.  There has been a PIP working at the care home providing this service in partnership with the GP practice to residents for the last 6 months In this interview we would like your views on the service and in particular your thoughts on the implementation and acceptability of the service as well as your perceptions of your working relationship with the PIP. |
| --- | --- | --- |
|  | The digital recorder  1. **Stress confidentiality** 2. **Set ground rules** | Can I check that you read and understood the participant information for this call? I would like to highlight the confidentiality of everything you tell me, and specifically that:   - *the recording will be deleted after being transcribed* - *you won’t be identified individually in any report.* - *all information will be anonymised.* - *we will not tell anyone else including your employer organisation, what you tell us as an individual.* - *likewise no other individual e.g. GP, care home staff, resident will be identifiable in any report.*   *We do need to remind you, however, that* if you do disclose anything which might identify a risk to yourself or to others, or a personal or professional offence, this would be shared with the relevant responsible authority. However we would tell you if we thought this were the case. |
|  | Ask if there are any questions |  |
|  | **Confirm consent** |  |

|  | **Stem question** | | | **Probes / follow ups** |
| --- | --- | --- | --- | --- |
| Part 1 | Views of the intervention | | |  |
| 1. | What do you know about the PIP service? | | |  |
| 2. | What involvement have you had with the PIP service? | | |  |
| 3. | Overall what are your views of the PIP service? | | | - New contributions - New problems |
|  |  | | Views on changing skill mix in the primary care team  Views on changing skills mix in the care home team | - Diversity in team(s) - Challenges - Issues in managing |
| 4. | How has the PIP service impacted on your work? (positively/negatively) | | | - Time - Referrals - Tests - Medication changes - Medication reviews - Repeat prescriptions - Medication administration processes - Record keeping - Medication storage - Interactions with GPs - Interaction with community pharmacist |
| 5. | In what ways has the PIP service affected patient care? (positively/negatively) | | | - Examples - Acceptability for patients without capacity? |
| Part 2 | Working relationship | | |  |
| 6. | What is your perception of your working relationship with the PIP | | | - Good relationship areas - Difficult relationship areas |
|  |  | | *How can your working relationship with the PIP be improved?* |  |
|  | **Stem question** | | | **Probes / follow ups** |
| 7. | How has your relationship with the PIP affected your relationship with your patients? | | | - New contributions - New problems |
| 8. | Where did communication issues arise between your and the other team members involved in the PIP service? | | | - PIP - Community Pharmacist - Primary care Pharmacist - District Nurse - GP Practice |
| 9. | How do you think communication impacted on patient care?  (positively/ negatively) | | | - Examples |
| Part 3 | Acceptability | | |  |
| 10. | What aspects of the PIP service went well from your perspective? | | | - Time commitment - Paper work - Issues raised/resolved |
| 11. | What aspects of the PIP service went less well from your perspective? | | | - Time commitment - Paper work - Issues raised/resolved |
|  |  | *How could we improve these?* | |  |
| 12. | What elements of the service did you like best? | | | - Patient care |
| 13. | Overall, how satisfied are you with the service provided by the PIP? | | |  |
| 14. | Would you like the service to continue? | | | - In its current from - In a revised form - Not at all |
| 15. | Any final comments | | |  |
| 16. | Thank you for taking part in this interview | | |  |

# **CHIPPS WP6 QUAL Evaluation: Topic Guide for stakeholder (GP) interview at 6 Months**

As the purpose of this interview is to encourage a conversation in which the participant can feel confident in expressing their own views, the following topic guide is indicative. This means that the interviewer may adapt it to suit the conversation style and preferences of the participant.

|  | Introduction.   - 1. **Introduce yourself**   2. **Explain the purpose of the research** | We have developed a new service in which a specially trained ‘pharmacist independent prescriber’ (PIP) becomes part of the care home team, working alongside general practitioners. The aim is to improve how resident’s medicines are managed in a safe, effective and cost effective way. We hope this may improve resident’s care, wellbeing and health outcomes. We are evaluating this new service in a large multi-centre randomised controlled trial in different places in the United Kingdom.  The study is run by the universities of East Anglia, Aberdeen, Leeds and Queen's Belfast. It is funded by the UK National Institute for Health Research.  You have had a PIP working with you and your team providing this service to your patients who are care home residents for the last 6 months. In this interview we would like your views on the service and in particular your thoughts on the implementation and acceptability of the service as well as your perceptions of your working relationship with the PIP. |
| --- | --- | --- |
|  | The digital recorder  1. **Stress confidentiality** 2. **Set ground rules** | Can I check that you read and understood the participant information for this interview? I would like to highlight the confidentiality of everything you tell me and specifically that:   - *the recording will be deleted after being transcribed* - *you won’t be identified individually in any report.* - *all information will be anonymised.* - *no other individual e.g. GP, Care Home staff or resident will be identifiable in any report* - *we will not tell anyone else including your employer organisation, what you tell us, as individuals*   *We do need to remind you, however, that* if you do disclose anything which might identify a risk to yourself or to others, or a personal or professional offence, this would be shared with the relevant responsible authority. However we would tell you if we thought this were the case.   - All your views are of value to us and there are no right or wrong answers. |
|  | Ask if there are any questions |  |
|  | **Confirm consent** |  |

|  | **Stem question** | | | **Probes / follow ups** |
| --- | --- | --- | --- | --- |
| Part 1 | Intervention | | |  |
| 1. | Overall what are your views of the PIP service? | | | - New contributions - New problems |
|  |  | | Views on changing skill mix in the primary care team  Views on changing skills mix in the care home team | - Diversity in team(s) - Challenges - Issues in managing |
| 2. | How has the PIP service impacted on your work load? | | | - (Negatively/ positively) - Time - Visit frequency - Referrals - Tests - Medication changes - Medication reviews - Medication ordering - Repeat prescriptions |
| 3. | In what ways has the PIP service impacted on patient care? | | | - Examples - Acceptability for patients without capacity? |
| Part 2 | Implementation | | |  |
| 4. | What barriers if any to implementing this service? | | |  |
|  |  | How could we overcome any barriers? | |  |
| 5. | What did you see as facilitating the service to be implemented? | | |  |
|  | **Stem question** | | | **Probes / follow ups** |
| Part 3 | Working relationship | | |  |
| 6. | What is your view of your working relationship with the PIP? | | | - Good relationship areas - Difficult relationship areas |
| 7. | Could the working relationship with the PIP have been improved? | | |  |
| 8. | How has the new service affected your relationship with your patients? | | | - New contributions - New problems |
| 9. | How has the new service affected your relationship with the care home staff? | | | - New contributions - New problems |
| 10. | Where did communication issues arise between you and the other team members of the team involved in the PIP service, and what were they? | | | - PIP - Care Home - Community Pharmacist - Primary care Pharmacist - District Nurse - GP Practice |
| 11. | How do you think any communication issues may have affected patient care? | | | - (Positively/ negatively) - examples |
| Part 4 | Acceptability | | |  |
| 12. | What aspects of the PIP service went well from your perspective? | | | - Time commitment - Paper work - Issues raised/resolved |
| 13. | What aspects of the PIP service went less well from your perspective? | | | - Time commitment - Paper work - Issues raised/resolved |
|  |  | How could we improve these? | |  |
| 14. | What elements of the service did you like best? | | | - Patient care |
| 15. | Overall, how satisfied are you with the service provided by the PIP? | | |  |
| 16. | Would you like the service to continue? | | | - In its current from - In a revised form - Not at all |
| 17. | Any final comments? | | |  |
| 18. | Thank you for taking part in this interview | | | |

# **CHIPPS WP6 QUAL Evaluation: Topic guide for stakeholder (residents/relatives) interview at 6 months**

As the purpose of this interview is to encourage a conversation in which the participant can feel confident in expressing their own views, the following topic guide is indicative. This means that the interviewer may adapt it to suit the conversation style and preferences of the participant.

|  | Introduction.   - 1. **Introduce yourself**   2. **Explain the purpose of the research** | We have developed a new service in which a specially trained ‘pharmacist independent prescriber’ (PIP) becomes part of the care home team, working alongside general practitioners. The aim is to improve how resident’s medicines are managed in a safe, effective and cost effective way. We hope this may improve resident’s care, wellbeing and health outcomes. We are evaluating this new service in a large study in different places in the United Kingdom.  The study is run by the universities of East Anglia, Aberdeen, Leeds and Queen's Belfast. It is funded by the UK National Institute for Health Research.  There has been a PIP providing this service in partnership with the GP practice to residents at this care home for the last 6 months. You/your relative have/has received this service. In this interview we would like your views on the service and in particular your thoughts on the implementation and acceptability of the service as well as your perceptions of your working relationship with the PIP. |
| --- | --- | --- |
|  | The digital recorder  1. **Stress confidentiality** 2. **Set ground rules** | Can I check that you remember you agreed (on behalf of your relative) to participate in the service and that you have read and understood the participant information for this interview?  I would like to highlight the confidentiality of everything you tell me and specifically that:   - *the recording will be deleted after being transcribed* - *you won’t be identified individually in any report.* - *all information will be anonymised.* - *no other individual e.g. GP, Care Home staff or resident will be identifiable in any report* - *we will not tell anyone else including your employer organisation, what you tell us, as individuals*   *We do need to remind you, however, that* if you do disclose anything which might identify a risk to yourself or to others, or a personal or professional offence, this would be shared with the relevant responsible authority. However we would tell you if we thought this were the case.   - All your views are of value to us and there are no right or wrong answers. |
|  | Ask if there are any questions |  |
|  | **Confirm consent** |  |

|  | **Stem question** | | | **Probes / follow ups** |
| --- | --- | --- | --- | --- |
| Part 1 Awareness of the intervention | | | | |
| 1. | Overall have you noticed any changes in your care/ the care of your relative/friend since the introduction of the PIP service? | | |  |
|  |  | What changes have you noticed? | | - Good/Bad - Examples |
| Part 2 Implementation | | | | |
| 2. | Before the PIP service started, what did you think about the information you received describing the new service? | | | - Relevant to you - Amount of detail - Understandable |
| 3. | Given information you had before the PIP service started, how far was the service what you thought it would be? | | | - Examples |
| Part 3 Relationship with PIP | | | | |
| 4. | How did you get on with your PIP? | | | - Good relationship areas - Difficult relationship areas |
|  |  | | *How could your relationship with the PIP be improved?* |  |
| 5. | How easy did you find it to talk to the PIP? | | | - Examples |
| 6. | How do you think your relationship with the PIP affect your care/ your relative/friends care? | | | - Examples |
| Part 4 Acceptability | | | | |
| 7. | What aspects of the PIP service went well from your perspective? | | | - Examples |
| 8. | What aspects of the PIP service went less well from your perspective? | | | - Examples |
| 9. | Is there anything we could improve the overall service? | | | - Examples |
| 10. | Overall how satisfied are you with the service provided by the PIP? | | |  |
| Part 5 | Study procedures | | |  |
| 16. | Recruitment and participation | | | - Acceptability for residents without capacity |
| 11. | Any final comments? | | |  |
| 12. | Thank you for taking part in this interview | | |  |

# **CHIPPS WP6 QUAL Evaluation: Topic Guide for Mentor interview**

As the purpose of this interview is to encourage a conversation in which the participant can feel confident in expressing their own views, the following topic guide is indicative. This means that the interviewer may adapt it to suit the conversation style and preferences of the participant.

|  | Introduction.   - 1. **Introduce yourself**   2. **Explain the purpose of the research** | The purpose of the CHIPPS study is to determine whether a PIP can improve the health outcomes and wellbeing of care home residents, by ensuring medicines are prescribed and managed in a safe, effective and cost effective way.  In this interview we would like to explore your views on CHIPPS, your role and experience as a mentor and your relationship with the PIP. |
| --- | --- | --- |
|  | The digital recorder  1. **Stress confidentiality** 2. **Set ground rules** | Can I check that you read and understood the participant information for this interview? I would like to highlight the confidentiality of everything you tell me and specifically that:   - *the recording will be deleted after being transcribed* - *you won’t be identified individually in any report.* - *all information will be anonymised.* - *no other individual e.g. GP, Care Home staff or resident will be identifiable in any report* - *we will not tell anyone else including your employer organisation, what you tell us, as individuals*   *We do need to remind you, however, that* if you do disclose anything which might identify a risk to yourself or to others, or a personal or professional offence, this would be shared with the relevant responsible authority. However we would tell you if we thought this were the case.  All your views are of value to us and there are no right or wrong answers. |
|  | Ask if there are any questions |  |
|  | **Confirm consent** |  |

|  | **Stem question** | | | **Probes / follow ups** |
| --- | --- | --- | --- | --- |
|  | Intervention | | |  |
| 1. | Overall what are your views of the PIP service? | | | - New contributions - New problems |
|  |  | | Views on changing skill mix in the primary care team  Views on changing skills mix in the care home team | - Diversity in team(s) - Challenges - Issues in managing |
| 2. | In general, what was your experience of providing a mentorship role for one of the PIPs involved in the study? | | | - How well-equipped to meet the mentorship role - Novel issues addressed - Examples of issues discussed - Ways in which mentor helped PIP |
| 3. | What was your experience of developing the personal development plan with the PIP? | | | - What are the benefits   - - To PIP?     - To the service-patients/GPs/care home staff? - Were there any barriers? - Would anything have made it easier? |
| 4. | What do you think about the competency framework assessment? | | | - Anything missing? - Hard/easy to demonstrate achievement - Any problems |
| 5. | What barriers if any to performing your role as a mentor? | | | - Barriers overcome - Barriers that could not be overcome |
| 6. |  | If yes, how did you address these barriers? Or how could they be overcome? | |  |
| 7. | Was there anything which facilitated your role as a mentor? | | |  |
| 8. | How often did you meet/talk with your PIP? | | | - Communication issues - How meetings were organised |
|  | Mentorship role | | |  |
| 9 | What previous experience if any of providing a mentorship role? | | |  |
| 10. | How did you prepare for your role as a mentor? | | | - Anything that proved useful - Any training CHIPPS should have provided? |
|  | Relationship with PIP | | |  |
| 11. | Your view of your relationship with the PIP? | | | - Good relationship areas - Difficult relationship areas |
|  |  | | |  |
| 12. | How could your relationship with the PIP be improved? | | |  |
|  | Would you do this PIP mentorship again? | | |  |
|  | Any final comments? | | | - PIP mentorship a useful role? |

1. A Pharmaceutical care plan is defined as plan for the responsible provision of medicine-related care for the purpose of achieving defined outcomes that improve the patient’s quality of life. It involves gathering information, identifying problems, assessing problems and desired improvements. [↑](#footnote-ref-1)
2. NCC MERP Taxonomy of Medical Errors. Available from: http://www.nccmerp.org/pdf/taxo2001-07-31.pdf [↑](#footnote-ref-2)
